# Association of Neural Activities in Language Processing and Memory with Rapid Reading

**DOI:** 10.14789/jmj.JMJ23-0022-OA

**Published:** 2023-11-29

**Authors:** YUYA SAITO, SEINA YOSHIDA, RYO UEDA, ATSUSHI SENOO

**Affiliations:** 1 Department of Radiology, Juntendo University Graduate School of Medicine, Tokyo, Japan; 1 Department of Radiology, Juntendo University Graduate School of Medicine, Tokyo, Japan; 2 Department of Radiological Sciences, Graduate School of Human Health Sciences, Tokyo Metropolitan University, Tokyo, Japan; 2 Department of Radiological Sciences, Graduate School of Human Health Sciences, Tokyo Metropolitan University, Tokyo, Japan; 3 Office of Radiation Technology, Keio University Hospital, Tokyo, Japan; 3 Office of Radiation Technology, Keio University Hospital, Tokyo, Japan

**Keywords:** memory, MRI, neural activity, language processing, rapid reading

## Abstract

**Objectives:**

To elucidate physiological changes in the brain caused by rapid reading, we herein focused on brain areas related to language processing and reading comprehension and memory processes and evaluated changes in neural activities associated with reading speed and comprehension using functional magnetic resonance imaging (fMRI).

**Materials:**

This study included 23 nonrapid and 23 rapid readers matched for age, gender, and handedness. T1 weighted image and fMRI were acquired using 3T MRI.

**Methods:**

The neural activity was compared between nonrapid and rapid readers using fMRI. The correlation between neural activity and reading speed and comprehension was also determined.

**Results:**

The neural activities of rapid readers were significantly lower in Wernicke's and Broca's areas, left angular and supramarginal gyri, and hippocampus. Furthermore, reading speed was negatively correlated with neural activities in these areas. Conversely, reading comprehension was negatively correlated with the neural activities in the left angular gyrus.

**Conclusions:**

Rapid readers exhibited reduced language processing, including phonological transformation, analysis, inner speech, semantic and syntactic processes, and constant reading comprehension during rapid reading.

## Introduction

Rapid reading is a way of efficiently obtaining information or knowledge by reading something quickly and is considered beneficial^1)^. Although it is believed that rapid reading worsens reading comprehension, a previous study showed that rapid readers had superior reading comprehension than nonrapid readers^2)^. Williams et al.^3)^ also found that students with faster reading skills had higher university grades than those with slower reading skills. Moreover, a study comparing rapid and nonrapid readers reported that the faster the reading speed, the better the reading comprehension^4)^. Furthermore, a study evaluating reading comprehension after rapid reading training revealed that reading comprehension did not decline compared with that before the training. However, the participants could read the sentences rapidly after the training^5)^, highlighting the advantages of rapid reading.

Despite the benefits of rapid reading, it is associated with distrust, which might be a major factor limiting its practice^6)^. According to Morita et al.^7)^, the phenomenon or mechanism underlying rapid reading is not well understood. Kurita^8-10)^ proposed the “super reading system (SRS),” which enables 80%-90% of the participants to improve their reading speed 10 times after approximately 50 h of training. However, the mechanism of acquiring rapid reading skills and the difference between normal and rapid reading have not been elucidated from the neuroscience perspective. To encourage rapid reading and derive benefits from it, establishing the scientific basis of physiological changes in the brain during rapid reading is necessary.

Although investigating physiological changes in the brain during rapid reading is challenging because of the scarcity of rapid readers, Fujimaki et al.^11, 12)^ explored these changes using functional magnetic resonance imaging (fMRI), which reflects neural activities indirectly using blood oxygenation level-dependent (BOLD) imaging. Rapid readers showed lower neural activities than nonrapid readers in Wernicke's and Broca's areas, which are associated with internal speech of articulation processes. This finding implies that rapid reading decreases or cuts the linguistic processes compared with ordinary reading. However, it remains unclear whether rapid readers understand the sentences they read or whether they exhibit improved reading comprehension^1-5)^. Moreover, previous studies had small sample sizes (rapid readers, n = 4 [1 male and 3 females]^11)^; n = 8 [1 male and 7 females]^12)^) and did not match age and sex between nonrapid and rapid readers. Therefore, to elucidate physiological changes in the brain caused by rapid reading, this study focused on brain areas related to language processing, such as Wernicke's and Broca's areas, supramarginal gyrus, and angular gyrus^11, 12)^, and reading comprehension and memory processes, such as the hippocampus^13-17)^, and evaluated changes in neural activities during rapid reading and comprehension in nonrapid and rapid readers using fMRI.

## Materials and Methods

### Participants

The study was approved by the Ethics Committee and Research and Development Department of Tokyo Metropolitan University (approval number: 22022). All participants provided written informed consent before participating in the study. Table 1 shows the participants' demographic data obtained at Tokyo Metropolitan University. This study included 23 nonrapid readers (age, 26.2 ± 9.5 years; male/female, 14/9; handedness, all right) and 23 rapid readers (age, 28.6 ± 7.8 years; male/female, 14/9; handedness, all right). To avoid the effect of age, sex, and handedness on the results, these parameters were matched between nonrapid and rapid readers.

### MRI acquisition

All MRI data were acquired using a 3-T scanner (Signa Premier; GE Healthcare, Chicago, IL, USA) with a 48-channel head coil. Whole-brain 3D magnetization prepared rapid gradient echo (MP-RAGE) T1-weighted imaging and T2*-weighted echo planar imaging, which are sensitive to the BOLD contrast and can reflect neural activities, were conducted in all participants. Whole-brain 3D MP- RAGE T1-weighted images were acquired using the following parameters: repetition time, 2210 ms; echo time, 3.24 ms; inversion time, 1000 ms; field of view, 220 × 220 mm; matrix size, 512 × 512; resolution, 0.43 × 0.43 mm; slice thickness, 1.0 mm; and acquisition time, 5 min 30 s. For the fMRI protocol, T2*-weighted echo planar diffusion-weighted images were acquired using spin-echo planar imaging with the following parameters: repetition time, 1000 ms; echo time, 30 ms; flip angle, 60°; field of view, 220 × 220 mm; matrix size, 64 × 64; resolution, 3.44 × 3.44 mm; slice thickness, 3.0 mm; and acquisition time, 9 min 40 s.

### fMRI tasks

**Figure 1:**
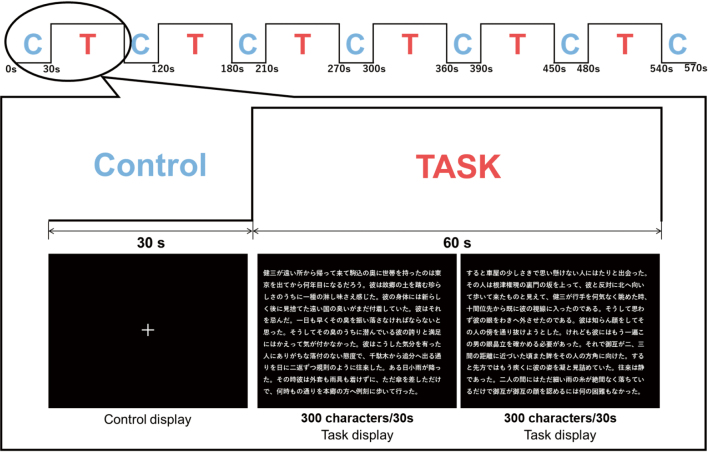
Task design in the fMRI session The protocol consisted of a block design that alternated the task block in 60 s and the control block in 30 s. In the task block of the fMRI session, several novels written by Natsume Soseki, a well-known Japanese novelist, were shown. In the task block, the participants were instructed to covertly read the sentences. In the rest block, a white cross was shown on a black background. The participants were instructed to look at the white cross without thinking to record only neural activities related to visual processes.

fMRI was conducted according to previously published methods (Figure 1)^11, 12)^. The fMRI protocol consisted of a block design that alternated the task block in 60 s and the control block in 30 s. In the task block of the fMRI session, several novels written by Natsume Soseki, a well-known Japanese novelist, were used: Kokoro (Heart), Higansugimade (To the spring equinox and beyond), Michikusa (Grass on the wayside), and Nowaki (Autumn wind) (Aozora-bunko; https://www.aozora.gr.jp/). In the task block, the participants were instructed to covertly read the sentences. In the rest block, a white cross was shown on a black background. The participants were instructed to look at the white cross without thinking to record only neural activities related to visual processes. Neural activities under the control conditions were subtracted from those under the test conditions to exclude visual- related activities.

Each participant was given a different novel to read, which the participant had either not read or had read it several years before the experiment. The novels were written in a mixture of kanji, hiragana, and katakana characters (three Japanese writing systems: morphograms [first] and syllabograms [last two]), where the characters were arranged in 12 rows and 27 columns (approximately 300 characters), from top to bottom and from right to left, which is the usual style for Japanese novels printed in paperback.

The characters were presented on a screen, which was placed 1.6 m away from the participants. Their size represented a visual angle of 0.46 × 0.46°, which was slightly larger than the typical size when books are physically read in a hardcopy form. The luminance was 0.75 cd/m^2^ for the characters and 160 cd/m^2^ for the background.

### Other data

Reading speed was calculated as characters per minute (cpm) in the 30-min session outside MRI and just before MRI. The reading comprehension score in the fMRI task was calculated as the cosine similarity between the original sentence shown in the task session and the sentence written by the participant 30 min right after MRI acquisition. The cosine similarity was calculated using Python version 3.7.0 (https://www.python.org/) with the MeCab (http://taku910.github.io/mecab/) and scikit-learn (https://scikit-learn.org/stable/) libraries.

### MRI preprocessing

fMRI image preprocessing was performed using the CONN toolbox v.21.a (Functional Connectivity SPM Toolbox 2021, McGovern Institute for Brain Research, Massachusetts Institute of Technology, http://ww.nitrc.org/projects/conn) relying on the SPM 12 software package (Wellcome Trust Centre for Neuroimaging, University College London, United Kingdom, http://www.fil.ion.ucl.ac.uk/spm/software) implemented in MATLAB 2019b (MathWorks, Natick, MA). All preprocessing steps were performed following the default preprocessing pipeline for volume-based analyses^18)^. Preprocessing included the following steps: (1)realignment and unwarping, (2)slice-timing correction, (3)structural segmentation and normalization, and (4)outlier identification.

In brief, all volumes of an fMR image were realigned with the first volume to correct for motion. The realigned fMR images were slice time- corrected, followed by tissue segmentation (i.e., gray matter-/white matter-/cerebrospinal fluid- normalized masks were determined) and coregistration to a T1-weighted image. Outlier identification was performed using artifact detection tools, which compute regressors for outliers and movements (i.e., resulting in scrubbing parameters). Participant movement realignment and scrubbing parameters (using conservative settings for functional outlier detection settings, global signal z- value threshold, and subject motion of 0.5 mm) were regarded as first-level covariates. Quality assurance (QA) plots were visually inspected to detect other possible outliers (i.e., “QA_ValidScans,” “QA_MaxMotion,” and “QA_InvalidScans”) and an adequate match with Montreal Neurological Institute space and proper coregistration across participants. After anatomical and functional preprocessing, denoising was performed to define, explore, and remove possible confounders in the BOLD signal. In denoising, linear regression and band-pass (i.e., 0.01-0.1 Hz) filtering were used to remove unwanted motion, white matter, cerebrospinal fluid noise components, and physiological noise sources, thereby reducing spurious sources of variance in fMRI.

After separating the preprocessed fMR images into task and rest sessions and averaging each volume using automated anatomical labeling atlas 3^19)^ in Wernicke's area (i.e., superior and posterior temporal gyri), Broca's area (i.e., angular gyrus), supramarginal gyrus, angular gyrus, and left hippocampus, the relative change in signal intensity was calculated as follows:




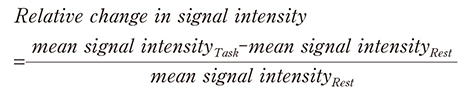




### Statistical analysis

All statistical analyses were performed using IBM SPSS Statistics for Windows, version 22.0 (IBM Corp., Armonk, NY, USA). Continuous data, such as reading speed, reading comprehension, and relative change in signal intensity, between nonrapid and rapid readers were compared using the Mann-Whitney U-test. In addition, to evaluate the relationship between neural activities associated with reading and reading skills (i.e., reading speed and comprehension), partial Spearman's rank correlation (*r_s_*) was calculated between them, with age and sex as the covariates. In all statistical analyses, false discovery rate (FDR) correction was used for multiple comparisons. A *P*-value of <.05 was considered statistically significant.

## Results

### Reading speed and comprehension

Table 1 shows the reading speed and comprehension scores of the nonrapid and rapid readers. Both the reading speed and comprehension scores were significantly higher among rapid readers than nonrapid readers (*P* < .001 and *P* < .05, respectively). Compared with nonrapid readers, the reading speed was higher by approximately 6.0 in rapid readers, whereas the reading comprehension score was higher by approximately 1.7 times.

**Table 1:** Participants' demographic data

	Nonrapid reader	Rapid reader	*P*
N	23	23	―
Age at baseline MRI, y	26.2 ± 9.5	28.6 ± 7.8	0.34
Sex, male/female	14/9	14/9	1.00
Handedness, L/R	0/23	0/23	1.00
Reading speed, characters per minute	720 ± 202	4191 ± 254	<.001
Reading comprehension score	27.8 ± 13.2	46.8 ± 7.1	<.05

Abbreviations: MRI, magnetic resonance imaging.

### Comparison of neural activities

**Figure 2:**
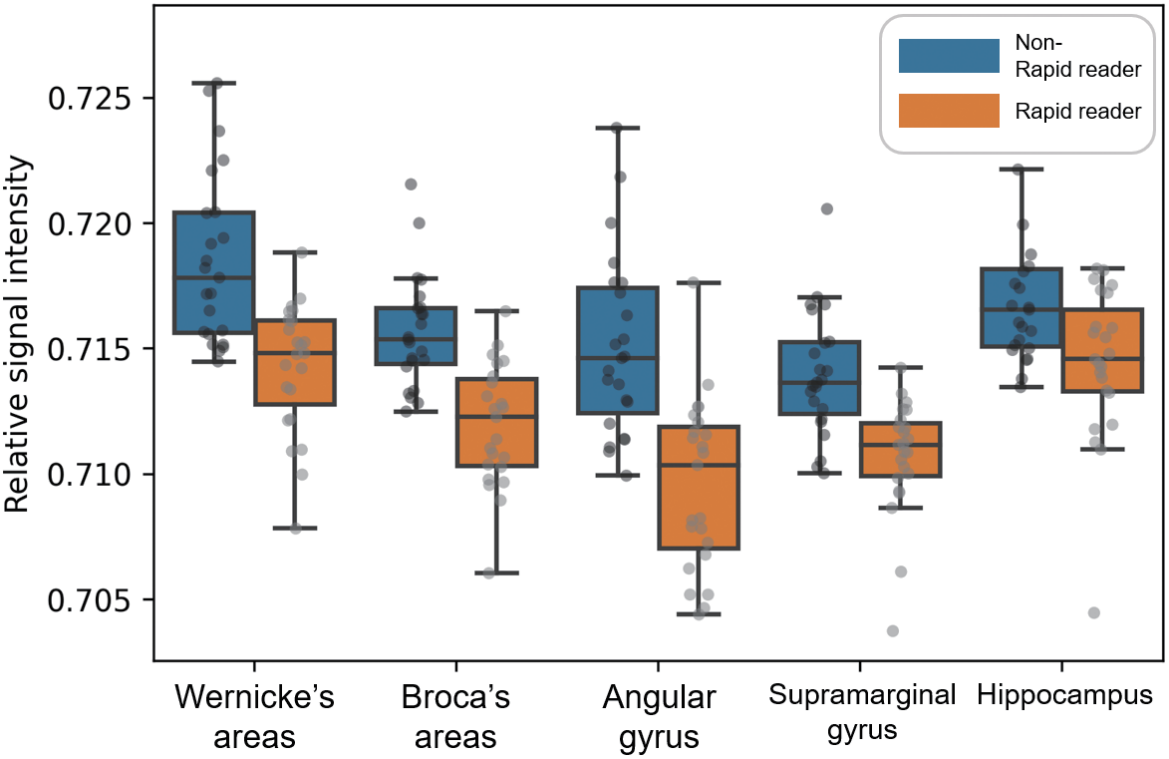
Comparison of neural activities Boxplots indicate the difference in relative signal intensity (i.e., neural activity) between nonrapid (blue) and rapid readers (red). The boxplots represent interquartile ranges, which contain 50% of the values of the participants. The whiskers are lines that extend from the box to the highest and lowest values. Neural activities of rapid readers were significantly lower in Wernicke’s (FDR-corrected *P* < .001) and Broca’s (FDR-corrected *P* < .001) areas, left angular (FDR-corrected *P* < .001) and supramarginal gyri (FDR-corrected *P* < .001), and hippocampus (FDR-corrected *P* < .01).

Table 2 and Figure 2 show the comparison of neural activities in each brain region between nonrapid and rapid readers. The neural activities of rapid readers were significantly lower in Wernicke's (FDR-corrected *P* < .001) and Broca's areas (FDR- corrected *P* < .001), left angular (FDR-corrected *P* < .001) and supramarginal (FDR-corrected *P* < .001) gyri, and hippocampus (FDR-corrected *P* < .01).

**Table 2:** Comparison of neural activities

	Nonrapid reader	Rapid reader	*FDR-corrected P*
Wernicke area	0.718 ± 0.003	0.714 ± 0.003	<0.001
Broca’s area	0.716 ± 0.002	0.712 ± 0.002	<0.001
Angular gyrus	0.714 ± 0.002	0.711 ± 0.002	<0.001
Supramarginal gyrus	0.715 ± 0.004	0.710 ± 0.003	<0.001
Hippocampus	0.717 ± 0.003	0.714 ± 0.003	<0.01

### Correlation between the change in neural activities and reading skills

**Figure 3:**
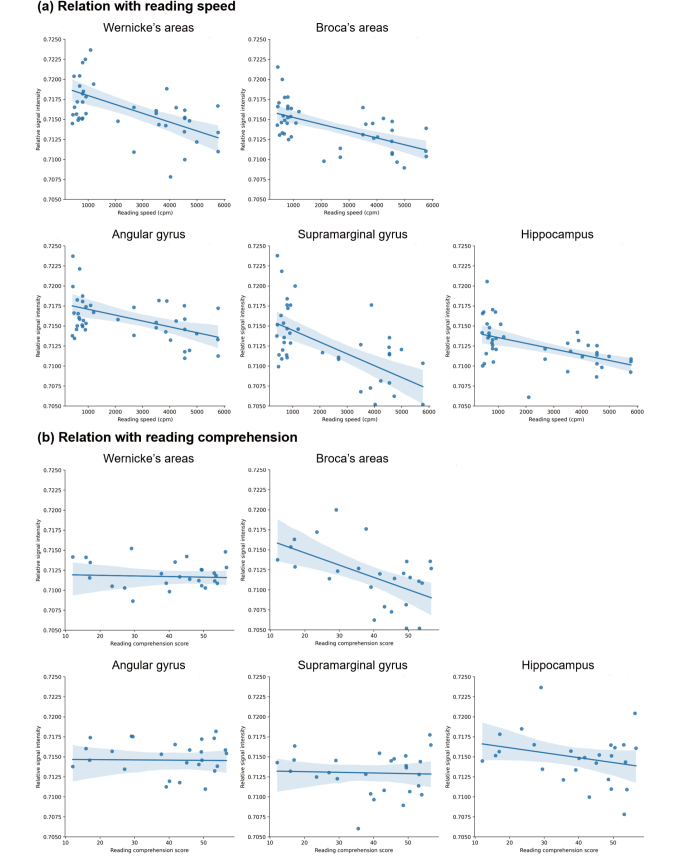
Scatter plot of the relationship between neural activities and reading skills Lines are estimated using linear regression, and error bar indicates 95% confidence interval. (a) Reading speed negatively correlated with neural activities in Wernicke’s (*r_s_* = −0.48, FDR-corrected *P* < .05) and Broca’s (*r_s_* = −0.68, FDR-corrected *P* < .001) areas, left angular (*r_s_* = −0.52, FDR-corrected *P* < .001) and supramarginal (*r_s_* = −0.53, FDR-corrected *P* < .001) gyri, and hippocampus (*r_s_* = −0.40, FDR-corrected P < .05). (b) On the contrary, reading comprehension negatively correlated with neural activities in the left angular gyrus (*r_s_* = −0.50, FDR-corrected *P* < .05).

Table 3 and Figure 3 show the correlation between neural activities in each brain region and reading skills, such as reading speed and comprehension. The reading speed was negatively correlated with neural activities in Wernicke's (*r_s_* = −0.48, FDR- corrected *P* < .05) and Broca's areas (*r_s_* = −0.68, FDR-corrected *P* < .001), left angular (*r_s_* = −0.52, FDR-corrected *P* < .001) and supramarginal gyri (*r_s_* = −0.53, FDR-corrected *P* < −.001), and hippocampus (*r_s_* = −0.40, FDR-corrected *P* < .05). In contrast, reading comprehension was negatively correlated with neural activities in the left angular gyrus (*r_s_* = −0.50, FDR-corrected *P* < .05).

**Table 3:** Correlation between neural activities and reading skills

	Neural activity vs. Reading speed		Neural activity vs. Reading comprehension score	
	*r_s_*	*FDR-corrected P*	*r_s_*	*FDR-corrected P*
Wernicke area	−0.48	<0.05	−0.18	0.55
Broca’s area	−0.60	<0.001	0.04	0.97
Angular gyrus	−0.52	<0.001	−0.50	<0.05
Supramarginal gyrus	−0.53	<0.001	0.01	0.97
Hippocampus	−0.40	<0.05	−0.01	0.97

## Discussion

To elucidate physiological changes in the brain during rapid reading, we herein focused on brain areas related to language processing, such as Wernicke's and Broca's areas, supramarginal gyrus, and angular gyrus, and reading comprehension and memory processes, such as the hippocampus, and evaluated changes in neural activities associated with reading speed and comprehension in nonrapid and rapid readers using fMRI. The results revealed that both the reading speed and comprehension scores were significantly higher in rapid readers than in nonrapid readers. Moreover, the neural activities of rapid readers were significantly lower in Wernicke's and Broca's areas, left angular and supramarginal gyri, and hippocampus. Furthermore, the reading speed was negatively correlated with neural activities in Wernicke's and Broca's area, left angular and supramarginal gyri, and hippocampus. Conversely, reading comprehension was negatively correlated with neural activities in the left angular gyrus.

Fujimaki et al.^11, 12)^ reported that neural activation in Wernicke's and Broca's areas decreased during rapid reading. This result was consistent with that of our study. Previous character and word experiments have demonstrated that neural activation in Wernicke's and Broca's areas was related to phonological processes, including phonological transformation, analysis, and inner speech^20, 21)^ and that activation of these areas depended on the task that demands inner speech^22, 23)^. Furthermore, experimental evidence supported a connectionist model in which these areas constitute a neural network for a phonological loop of working memory^24)^. Thus, the result obtained in the present study that neural activities were significantly lower in rapid readers and negatively correlated with reading speed suggests that phonological processes, such as phonological transformation and accompanying inner speech, were reduced during rapid reading.

Since the early 20th century, damage to the left angular gyrus^25)^ and both the left angular and supramarginal gyrus^26)^, is associated with language comprehension deficits. Recently, fMRI studies have shown that these regions together with the left inferior parietal lobe are activated in language comprehension through semantic and syntactic processes^27-31)^. In the present study, neural activities in the left angular and supramarginal gyri were significantly lower in rapid readers and negatively correlated with reading speed, indicating that rapid readers had reduced semantic and syntactic processes while maintaining reading comprehension during rapid reading.

Regarding neural activities in the hippocampus, research on brain regions of humans and experimental animals has identified a system of hippocampal structures essential for the formation of learning and memory (e.g., reading contents)^32)^. Neuroimaging techniques, such as fMRI and positron emission tomography (PET), have provided additional evidence for the importance of the hippocampus in learning and memory^33, 34)^. Moreover, in a recognition memory study, Stark and Squire^35)^ found increased hippocampal activities during the retrieval of previously presented words or objects. Eldridge et al.^36)^ also reported that hippocampal activities during recognition memory for words are selective for episodic rather than nonepisodic retrieval. Interestingly, against all expectations, the present study indicated that neural activities in the left hippocampus significantly decreased in rapid readers and negatively correlated with the reading speed. Thus, a rapid reader exhibited reduced retrieval of previously presented words or objects while maintaining reading comprehension. This finding suggests that nonrapid and rapid readers have different reading strategies. Indeed, Douglas et al. reported that reading speed was more related to visual acuity during rapid reading^37)^. In addition, a study using fMRI showed increased neural activities with increasing reading speed in the right intraparietal sulcus, which is considered to reflect visuospatial processes^12)^. However, our study did not measure visual activity during reading, nor did it prove that rapid readers in this research exhibited better visual acuity. Therefore, further study including measurement of visual activity during reading is required.

In the present study, both reading speed and comprehension were significantly higher in rapid readers than in nonrapid readers. Stevens et al.^37)^ investigated reading speed and comprehension in college freshmen with nonrapid and rapid reading skills and reported that rapid readers read sentences with higher reading comprehension and speed. Our result was consistent with that of the abovementioned study by Stevens et al. in terms of the relationship between reading speed and comprehension. On the contrary, studies have reported that the activity level in the left hippocampus during learning positively correlated with subsequent recognition memory accuracy^38-40)^. The reading comprehension score did not significantly correlate with neural activities in the left hippocampus, whereas a significant negative correlation was observed between reading comprehension score and neural activities in the left angular gyrus. The aforementioned studies targeted nonrapid readers, whereas the present study included both rapid and nonrapid readers. Considering that the reading strategy might be different between nonrapid and rapid readers as mentioned above, an inconsistency in the correlation between neural activities in the left hippocampus and memory may arise between our results and those of previous studies. To elucidate this mechanism, further studies including brain network analysis are warranted to consider microstructural and structural connectivities using diffusion-weighted MRI and functional connectivity using fMRI.

This study had some limitations. First, the sample size was small. To improve the statistical power and reliability of the results, a larger sample size is required. However, collecting data on rapid readers is challenging because only a few individuals have rapid reading skills. Second, MRI data were obtained using a single scanner in a single institution. Further longitudinal and multicenter studies incorporating scanners from different sites with different field strengths and manufacturers would be beneficial for robust results that do not depend on the institution and scanner. Third, the fMRI task included only Japanese tasks. Therefore, it remains unclear whether our results make sense for other language or not, although language differs. Fourth, this was a cross-sectional study. Longitudinal studies are necessary to clarify physiological changes in the brain during rapid reading.

This study evaluated neural activities associated with reading speed and reading comprehension during rapid reading, which were not evaluated in previous studies. In addition, this study showed great consistency with previous studies, and rapid readers read sentences while maintaining reading comprehension.

In conclusion, neural activities of rapid readers were significantly lower in Wernicke's and Broca's areas, left angular and supramarginal gyri, and hippocampus. Furthermore, reading speed was negatively correlated with neural activities in Wernicke's and Broca's area, left angular and supramarginal gyri, and hippocampus. On the contrary, reading comprehension was negatively correlated with neural activities in the left angular gyrus. These results suggest that rapid readers have reduced language processes, such as phonological processes (e.g., phonological transformation, analysis, and inner speech) and semantic and syntactic processes, and constant reading comprehension during rapid reading. The findings of this study could deepen our understanding of the mechanism of rapid reading and provide a scientific basis for physiological changes in the brain during rapid reading, thereby reducing distrust toward rapid reading and promoting benefit from it.

## Funding

This research was supported by Tokyo Metropolitan University Graded Research Funds (Departmental Competitive Funds) 2021.

## Author contributions

YS, SY, and AS conceived the presented idea. YS developed the theory and investigated the report on this research. AS and RU encouraged YS to investigate this work. All authors discussed the results and contributed to the final manuscript.

## Conflicts of interest statement

All authors declare that the research was conducted without any commercial or financial relationships that could be construed as a potential conflict of interest.
